# Supplementary material for: Modulating Heterologous Pathways and Optimizing Culture Conditions for Biosynthesis of trans-10, cis-12 Conjugated Linoleic Acid in Yarrowia lipolytica
Source: Molecules. 2019 May 6;24(9):1753. doi: 10.3390/molecules24091753 (PMC6539415; doi:10.3390/molecules24091753)

Table S1 Primers used for genetic modifications.

Name	Description (Sequence5′ → 3′)	
GPD1-F	CGGGGTACCCTAGTTGGCGTGGTAAAGAATCTC	pWX015
GPD1-R	CACGTGATGAGCGCTCTACTTCGATCG	pWX015
DGAT-F	CGGGGTACCTTACTCAATCATTCGGAACTC	pWX010
DGAT-R	CACGTGATGACTATCGACTCACAATACTAC	pWX010
MA12D-RHF	AGATTCCGGCCTCTTCGGCCGCCACCATGGCTCCACCTAACAC	pWX020, pWX034
MA12D-RHR	GGACAGGCCATGGAGGTACCTTACTTCTTAAAGAACACAACATCGCC	pWX020, pWX034
1267-RHF	GGTACCTCCATGGCCTGTCCC	pWX020
1267-RHR	GGCCGAAGAGGCCGGAATC	pWX020
1267MA12D-F	ACGGGCATCTCACTTGCGTA	pWX025
1267MA12D-R	GTCCGAATTCCATGTGTAACAC	pWX025
DGAT-RHF	TACACATGGAATTCGGACGAATTCGGACACGGGCATCT	pWX025, pWX037
DGAT-RHR	CGCAAGTGAGATGCCCGTGGTTGAGGCCGTTGAGCACC	pWX025, pWX037
PAI-RHF	ACCACACACATCCACGTGATGTCTATCTCCAAAGACAGCCG	pWX030
PAI-RHR	ACAGGCCATGGAGGTACCTCAAACGAAGAAGCGGGTAACCA	pWX030
1312-RHF	GGTACCTCCATGGCCTGTCC	pWX030
1312-RHR	CACGTGGATGTGTGTGGTTGTA	pWX030
1312PAI-F	GTCCGAATTCCATGTGTAACAC	pWX034, pWX037
1312PAI-R	ACGGGCATCTCACTTGCGTA	pWX034, pWX037

Table S2 Genebank ID and sequences of enzymes used in this study.

Name	Amino acid sequence	Genebank ID
GPD1	msallrsslrfkhmsavnrltqqlrlltasaplsaantagkapfkvavvgsg nwgttvakivaenctahpelfepevrvwvreekvngknltdifnaehen vrylpkiklphnliaepdllkaveganiivfnlphqflagvckqlkghvnp karaisclkgldvtpqgvyllsdvienetglhcgvlsganlateialekyset tvaynrpkdffgegdvtndvlkalfhrpyfhvrcvqdvagvsiggalknv valcagfvegknwgdnakaaimrrgmleminfskrffpetdintltvesa gvadlitscaggrnfkvgrafgkesgsgktiqdvekellngqsaqgvitcn evhellknknmqkdfplfestwgiihgelkiddlpeilyhan	YALI0B02948p
DGAT	mtidsqyyksrdkndtapkiagiryaplstpllnrcetfslvwhifsiptflti fmlccaipllwpfviayvvyavkddspsnggvvkryspisrnffiwklfg ryfpitlhktvdlepthtyypldvqeyhliaerywpqnkylraiistieyflp afmkrslsineqeqpaerdpllspvspsspgsqpdkwinhdsrysrgess gsnghasgselngngnngttnrrplssasagstasdstllngslnsyanqii gendpqlsptklkptgrkyifgyhphgiigmgafggiategagwsklfpg ipvslmtltnnfrvplyreylmslgvasvskksckallkrnqsicivvgga qesllarpgvmdlvllkrkgfvrlgmevgnvalvpimafgendlydqvs ndkssklyrfqqfvknflgftlplmhargvfnydvglvpyrrpvnivvgs pidlpylphptdeevseyhdryiaelqriynehkdeyfidwteegkgape frmie	YALI0E32769g
MA12 D	mappntidagltqrhistsapnsakpafernyqlpeftikeirecipahcfer sglrglchvaidltwasllflaatqidkfenplirylawpvywimqgivctg vwvlahecghqsfstsktlnntvgwilhsmllvpyhswrishskhhkat ghmtkdqvfvpktrsqvglppkenaaaavqeedmsvhldeeapivtlf wmviqflfgwpaylimnasgqdygrwtshfhtyspifeprnffdiiisdl gvlaalgaliyasmqlslltvtkyyivpylfvnfwlvlitflqhtdpklphyr egawnfqrgalctvdrsfgkfldhmfhgivhthvahhlfsqmpfyhaee atyhlkkllgeyyvydpspivvavwrsfrecrfvedqgdvvffkk	KF667536
PAI	msiskdsriaiigagpaglaagmyleqagfhdytilertdhvggkchspn yhgrryemgaimgvpsydtiqeimdrtgdkvdgpklrreflhedgeiyv pekdpvrgpqvmaavqklgqllatkyqgydanghynkvhedlmlpfd eflalngceaardlwinpftafgyghfdnvpaayvlkyldfvtmmsfak gdlwtwadgtqamfehlnatlehpaernvditritredgkvhihttdwdr esdvlvltvplekfldysdaddereyfskiihqqymvdaclvkeyptis gyvpdnmrperlghvmvyyhrwaddphqiittyllrnhpdyadktqee crqmvlddmetfghpvekiieeqtwyyfphvssedykagwyekveg mqgrrntfyageimsfgnfdevchyskdlvtrffv	AX062088

GPD1: glycerol-3-phosphate dehydrogenase

DGA1: diacyl-glycerol transferase

MA12D: Δ12 desaturase

PAI: linoleate isomerase

Figure S1 All recombinant plasmid maps utilized in this work.

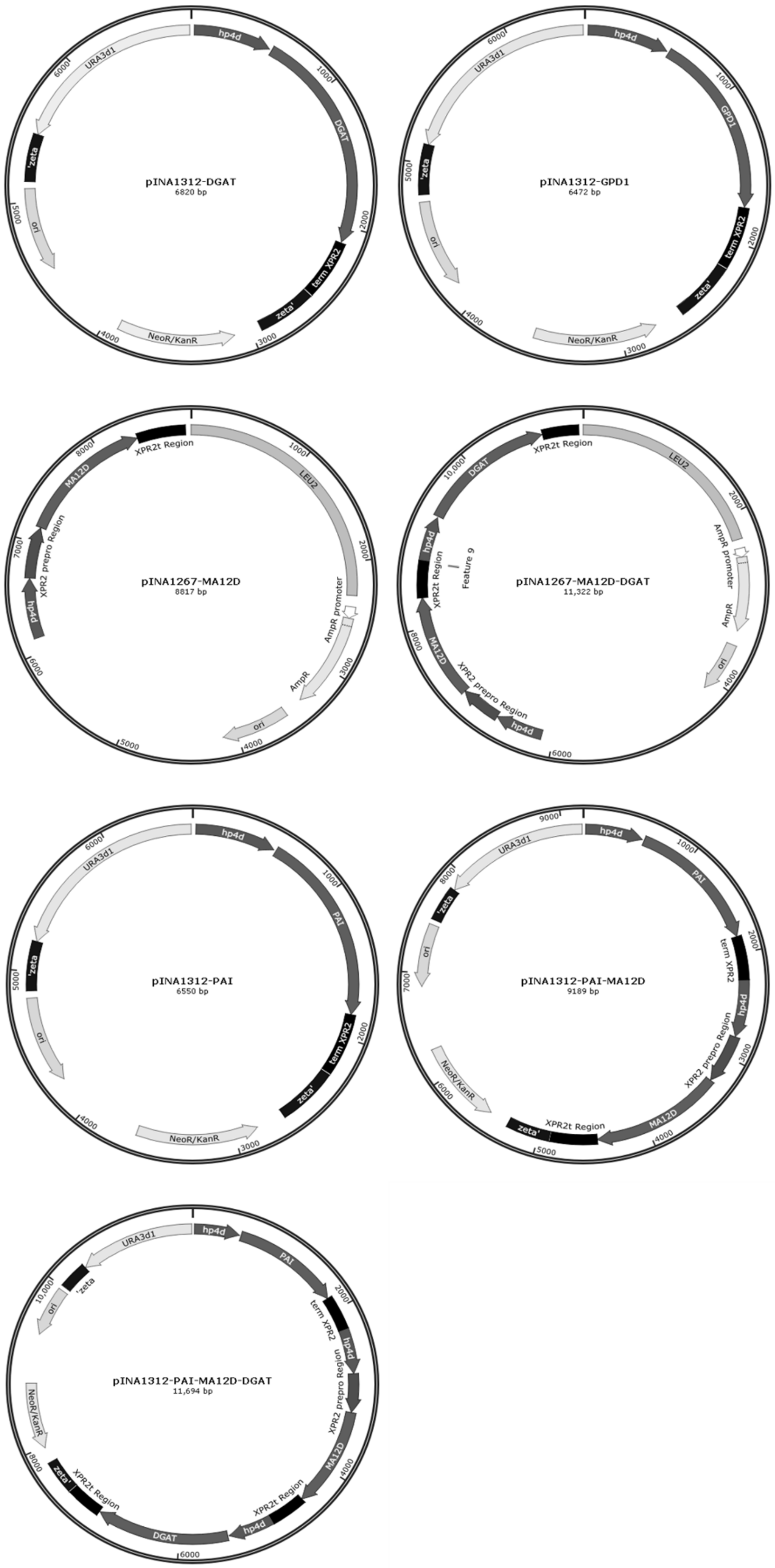

Figure S2 Nile red dying of wild strains (A) and engineered strains (B).

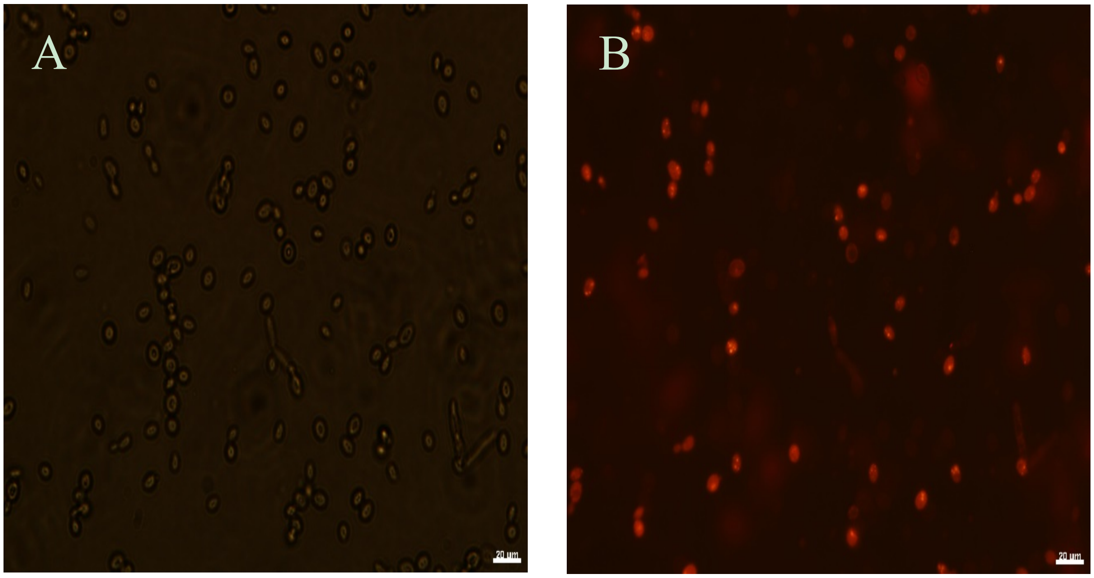

Figure S3 Gas chromatogram analysis of FAMEs (a mixture of 1. methyl palmitate, 2. methyl palmitoleate, 3. methyl stearate, 4. methyl oleate, 5. methyl linoleate and 6. conjugated linoleic acid methyl ester) from transformed yeasts. (a) FAMEs from wild strain *Y. lipolytica* ATCC20460 (b) FAMEs from recombinant pWX020 (c) FAMEs from recombinant pWX030 (d) FAMEs from recombinant pWX037.

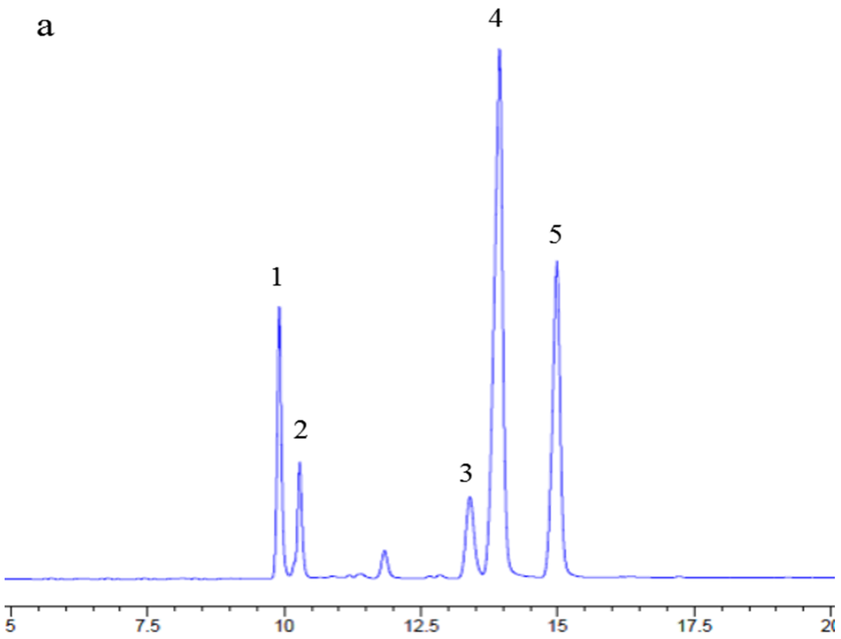

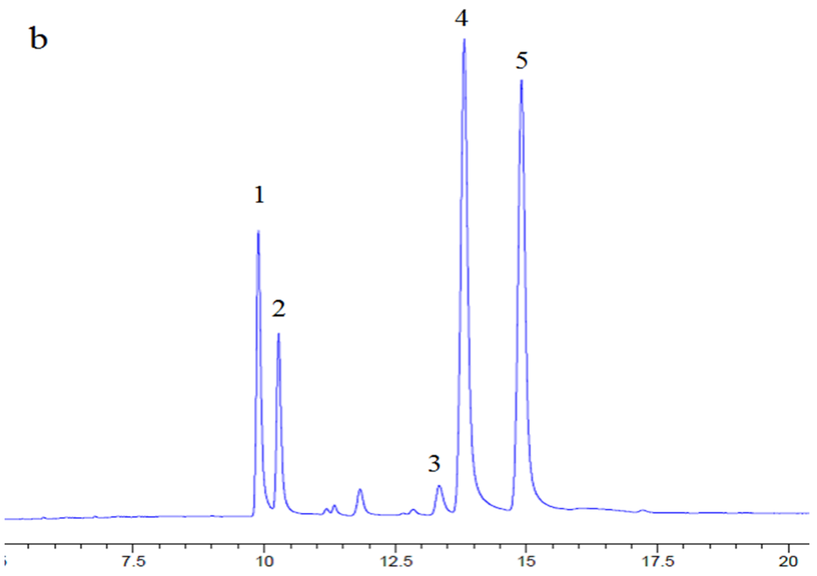

c

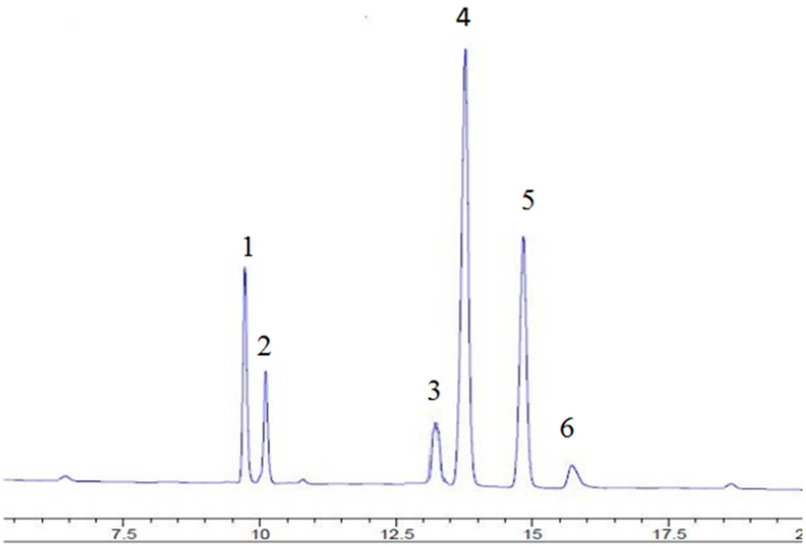

d

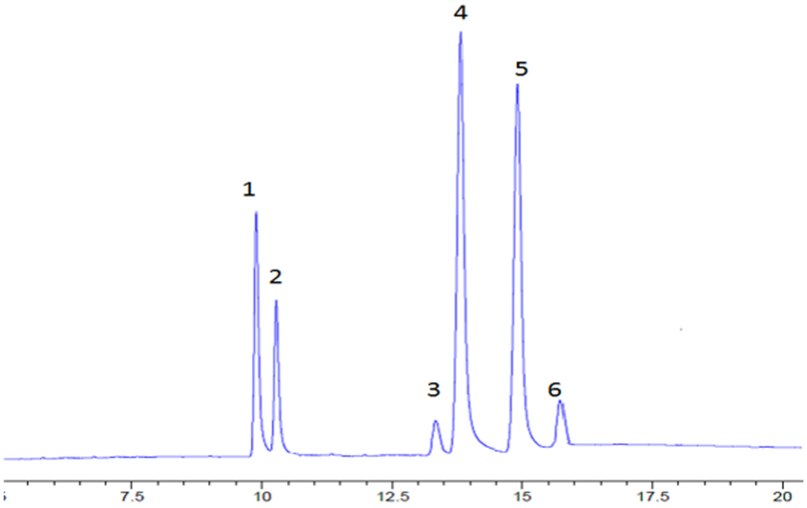

Figure S4 The structure of various fatty acids. (a) palmitic acid (b) palmitoleate acid (c) stearic acid (d) oleic acid (e) conjugated linoleic acid

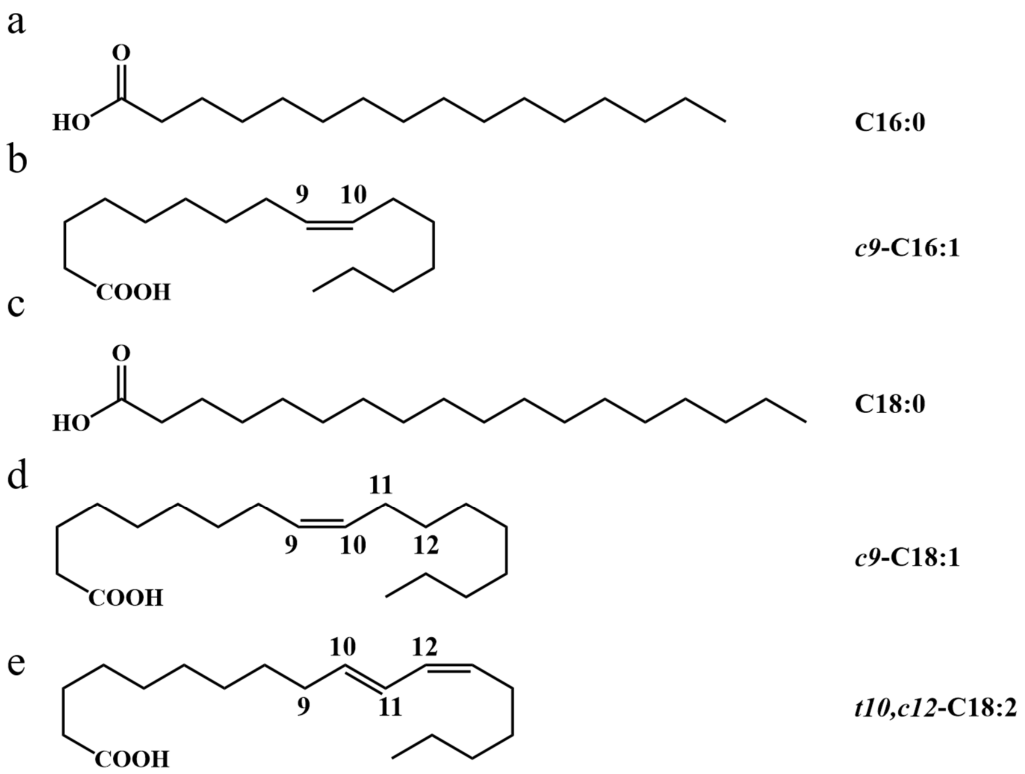

Supplement: Supplementary file 1 [file molecules-24-01753-s001.pdf]
